# Supplementary material for: Attendee’s awareness about preventive chemotherapy neglected tropical diseases (PC-NTD) control during the first world neglected tropical diseases day in Ekiti State, Nigeria
Source: PLoS Negl Trop Dis. 2021 Mar 29;15(3):e0009315. doi: 10.1371/journal.pntd.0009315 (PMC8032117; doi:10.1371/journal.pntd.0009315)
Supplement: S1 STROBE Checklist — (DOC) [file pntd.0009315.s001.doc]

STROBE Statement—Checklist of items that should be included in reports of ***cross-sectional studies***

|  | Item No | Recommendation |
| --- | --- | --- |
| **Title and abstract** | 1 | (*a*) the word during in the title shows the study is cross-sectional in design |
|  |  | (*b*) Informative and balanced summary has been provided in the abstract, page 2 (Line 26-50) and Page 3 (Line 54-62) of the manuscipt |
| Introduction | | |
| Background/rationale | 2 | Introduction, Page 5(Line 75-103) section has enough scientific background and rationale , Page 5 (Line 105-112) for the study being carried out |
| Objectives | 3 | The later end of the introduction, has the specific objective for the research Page 5(Line 108-113) |
| Methods | | |
| Study design | 4 | The study design is clearly stated as early as possible, Page 6 (Line 125- 131) |
| Setting | 5 | The study settling and locations has been clearly described in Page 6 (Line 125-131) |
| Participants | 6 | Page 6-7 (Line 133-150) clearly describes the sources and method of selection of participants, as well as eligibility criteria |
| Variables | 7 | N/A |
| Data sources/ measurement | 8* | *Page 6 (Line141-147) and Page 7 (Line 148-150*) clearly describe the source and methods of assessment of stool samples in the study. |
| Bias | 9 | Information on how we prevented recording bias was provided on Page 7 (Line 148-150) |
| Study size | 10 | The method employed was clearly given in Page 7 (Line 143-145) |
| Quantitative variables | 11 | How quantitatve variables were handled was provided in Page 7 (Line 154-158) |
| Statistical methods | 12 | (*a*) the relevant information has been provided in Page 7 (Line 154-158) |
|  |  | (*b*) the relevant information has been provided in Page 7 (Line 154-158) |
|  |  | (*c*) N/A |
|  |  | (*d*) N/A |
|  |  | (*e*) N/A |
| Results | | |
| Participants | 13* | (a) N/A |
|  |  | (b) N/A |
|  |  | (c) N/A |
| Descriptive data | 14* | (a) Page 8 (Line 187-192) contains reports on demography of study participants |
|  |  | (b) N/A |
| Outcome data | 15* | Outcome events has been reported quantitatively on Page 8-14 (Line 201-334) |
| Main results | 16 | (a) Results has been reported quantitatively and qualitatively on Page 8-14 (Line 201-334) |
|  |  | (*b*) N/A |
|  |  | (*c*) N/A |
| Other analyses | 17 | N/A |
| Discussion | | |
| Key results | 18 | The summary of the key results in reference to study objectives have been discussed on Page 14-18 (Line 331-418) |
| Limitations | 19 | N/A |
| Interpretation | 20 | The interpretation of the result have been simultaneously discussed with the summary on Page 14-18 (Line 331-418) |
| Generalisability | 21 | Information on the general validity of the findings have been simultaeneously provided on on Page 14-18 (Line 331-418) |
| Other information | | |
| Funding | 22 | Funding details has been sufficiently described on line 439-444 |

*Give information separately for exposed and unexposed groups.

**Note:** An Explanation and Elaboration article discusses each checklist item and gives methodological background and published examples of transparent reporting. The STROBE checklist is best used in conjunction with this article (freely available on the Web sites of PLoS Medicine at http://www.plosmedicine.org/, Annals of Internal Medicine at http://www.annals.org/, and Epidemiology at http://www.epidem.com/). Information on the STROBE Initiative is available at www.strobe-statement.org.
