# Supplementary material for: Attendee’s awareness about preventive chemotherapy neglected tropical diseases (PC-NTD) control during the first world neglected tropical diseases day in Ekiti State, Nigeria
Source: PLoS Negl Trop Dis. 2021 Mar 29;15(3):e0009315. doi: 10.1371/journal.pntd.0009315 (PMC8032117; doi:10.1371/journal.pntd.0009315)
Supplement: S1 Text — (DOCX) [file pntd.0009315.s002.docx]

**Dear Attendee,**

Please kindly provide answers to the following questions to participate in a survey on public awareness and knowledge about NTDs

…………………………………………………………………………………………………………………………………

**Personal Information**

Age: (a) <15years (b) 15-25years (a) 26-35years (b) >35years

Gender: (a) Male (b) Female

Occupation: a) Student/unemployed (b) Academic (c) NTD programmer (d) Government official/policy maker (e) Media (f) Civil servants (g) Others, please specify …………………………..

Name of Institution:...................................................................................................................................

Name of Department: ................................................................................................................................

**Other questions**

1. Have you ever heard of NTDs before now? (a) Yes (b) No
2. If yes, which of the following is a NTDs;

(a) Malaria (b) Schistosomiasis (c) Tuberculosis (d) Lymphatic Filariasis (e) Diabetes (f) HIV (g) Onchocerciasis (h) Worm infections

1. Where did you first learn or hear about NTDs?

(a) Online radio program (b) school (c) hospital/clinic (d) This event advert, rally or radio program.

1. Please in a single sentence what do NTDs mean to you

…………………………………………………………………………………………………

1. Do you think NTDs is a public health problem? (a) Yes (b) No
2. Why do you think so

…………………………………………………………………………………………………

1. Have you seen anyone or do you know anyone affected by NTDs before? (a) Yes (b) No
2. Have you seen anyone or do you know anyone with swollen limbs and thighs? (a) Yes (b) No
3. Have you seen anyone or do you know anyone with bloody urine? (a) Yes (b) No
4. Have you seen anyone or do you know anyone with worm in stool? (a) Yes (b) No
5. Have you seen anyone or do you know anyone with de-pigmented skin? (a) Yes (b) No
6. Are you aware of the treatment programme going on in schools and communities? (a) Yes (b) No
7. Who do you think are in charge of this treatment programme?.......................................................
8. Where did you first learn about this treatment programme?

(a) Online radio program (b) hospital/clinic (c) This event advert, rally or radio program (d) Others, please specify ………………………………………….

1. Have you, or do you know anyone who have taken those drugs before? (a) Yes (b) No
2. If yes, what drug and where did you/they get the drugs from................................................................
3. Have you received health education messages regarding NTDs before? (a) Yes (b) No
4. If yes, where? .....................................................................
5. If yes, what message and where did they hear about NTDs...........................................................
6. Generally, do you think there is enough awareness about NTDs?
7. Will you wish to participate in any activity related to NTDs in the state (a) Yes (b) No
8. If yes, please tick your preference activities

(a) Advocacy and health promotion (b) Networking (c) Research

**Please submit this questionnaire and collect your materials**
