# Supplementary material for: Attendee’s awareness about preventive chemotherapy neglected tropical diseases (PC-NTD) control during the first world neglected tropical diseases day in Ekiti State, Nigeria
Source: PLoS Negl Trop Dis. 2021 Mar 29;15(3):e0009315. doi: 10.1371/journal.pntd.0009315 (PMC8032117; doi:10.1371/journal.pntd.0009315)
Supplement: Attachment — Submitted filename: PNTD-D-20-02136_reviewer (2)_ Jan 21_MPS comments.pdf [file pntd.0009315.s004.pdf]

PLOS Neglected Tropical Diseases

Attendee Awareness and Knowledge about Preventive Chemotherapy Neglected Tropical Diseases (PC-NTD) control during the First World Neglected Tropical Diseases Day in Ekiti State, Nigeria.

--Manuscript Draft--

**Manuscript Number:**	PNTD-D-20-02136
**Full Title:**	Attendee Awareness and Knowledge about Preventive Chemotherapy Neglected Tropical Diseases (PC-NTD) control during the First World Neglected Tropical Diseases Day in Ekiti State, Nigeria.
**Short Title:**	Attendee awareness during the First World NTD Day in Ekiti State, Nigeria
**Article Type:**	Research Article
**Keywords:**	World NTD Day; Awareness; Elimination; 2020; Control; Ekiti; Nigeria
**Abstract:**	Background<br><br>The need to control Neglected Tropical Diseases (NTDs) and sustain progress towards elimination through mass administration of medicines requires substantial communal participation. This study, therefore, assessed the knowledge and perception of attendees' regarding NTDs and its control activities during the Inaugural World NTD day event in Ekiti State, Nigeria.<br><br>Methodology<br><br>A cross-sectional study involving the administration of pretested semi-structured questionnaires to consenting attendees at the Inaugural World NTD day event was conducted on the 30 th January, 2020. The questionnaire collected data on attendee's demography, knowledge and awareness about NTDs in Nigeria. Quantitative data were analysed using descriptive statistics in SPSS. 20.0 Software and expressed as frequencies and percentages. However, qualitative data to support quantitative analysis were obtained using open-ended questionnaires and analysed thematically.<br><br>Principal findings/conclusion<br><br>A total of 309 attendees participated in this study comprising 167 (54.0%) females, and 142(46.0%) males. 167 (54.0%) of the attendees have not heard about NTDs before, whereas 77(24.92%) have heard about NTDs through the advertisement of the event. 105(33.98%) of the attendees were unaware of ongoing NTD control programs in schools and communities. Also, 246 79.61%) of them have not taken or do not know anyone that has taken drugs donated in schools or communities before. The number of attendees 41(13.3%) who incorrectly classified malaria as NTDs is higher than those who recognized onchocerciasis 36 (11.7%) and worm infections 34(11.0%) as NTDs (p>0.05). This study has shown that awareness and knowledge about NTDs control activities in Ekiti State is low, thus justifying the event as an awareness day for addressing NTDs. Public enlightment and regular promotional activities such as media engagement will raise the public appreciation and participation in NTDs control activities.
**Additional Information:**	
**Question**	**Response**
**Financial Disclosure**<br><br>Enter a financial disclosure statement that describes the sources of funding for the work included in this submission. Review the submission guidelines for detailed requirements. View published research	We express our deep appreciation to Crown Prince Court of Abu Dhabi for providing the micro-grant for the Inaugural World NTD day event in Ekiti State, Nigeria. However, funds for the research were provided by the first author, hence Crown Prince Court of Abu Dhabi had no role in funding this research, the study design, data collection and analysis, decision to publish, or preparation of the manuscript.

Powered by Editorial Manager® and ProduXion Manager® from Aries Systems Corporation

articles from *PLOS NTDs* for specific examples.<br><br>This statement is required for submission and **will appear in the published article** if the submission is accepted. Please make sure it is accurate.<br><br>**Unfunded studies**<br><br>Enter: *The author(s) received no specific funding for this work.*<br><br>**Funded studies**<br><br>Enter a statement with the following details:<br>• Initials of the authors who received each award<br>• Grant numbers awarded to each author<br>• The full name of each funder<br>• URL of each funder website<br>• Did the sponsors or funders play any role in the study design, data collection and analysis, decision to publish, or preparation of the manuscript?<br>• **NO** - Include this sentence at the end of your statement: *The funders had no role in study design, data collection and analysis, decision to publish, or preparation of the manuscript.*<br>• **YES** - Specify the role(s) played.<br><br>* typeset	
**Competing Interests**<br><br>Use the instructions below to enter a competing interest statement for this submission. On behalf of all authors, disclose any competing interests that could be perceived to bias this work—acknowledging all financial support and any other relevant financial or non-financial competing interests.	The authors have declared that no competing interests exist.

This statement **will appear in the published article** if the submission is accepted. Please make sure it is accurate. View published research articles from *PLOS NTDs* for specific examples.<br><br>**NO authors have competing interests**<br><br>Enter: *The authors have declared that no competing interests exist.*<br><br>**Authors with competing interests**<br><br>Enter competing interest details beginning with this statement:<br><br>*I have read the journal's policy and the authors of this manuscript have the following competing interests: [insert competing interests here]*<br><br>* typeset	
**Data Availability**<br><br>Authors are required to make all data underlying the findings described fully available, without restriction, and from the time of publication. PLOS allows rare exceptions to address legal and ethical concerns. See the PLOS Data Policy and FAQ for detailed information.<br><br>A Data Availability Statement describing where the data can be found is required at submission. Your answers to this question	Yes - all data are fully available without restriction

constitute the Data Availability Statement and **will be published in the article**, if accepted.<br><br>**Important:** Stating 'data available on request from the author' is not sufficient. If your data are only available upon request, select 'No' for the first question and explain your exceptional situation in the text box.<br><br>Do the authors confirm that all data underlying the findings described in their manuscript are fully available without restriction?	
**Describe where the data may be found in full sentences. If you are copying our sample text, replace any instances of XXX with the appropriate details.**<br><br>• If the data are **held or will be held in a public repository**, include URLs, accession numbers or DOIs. If this information will only be available after acceptance, indicate this by ticking the box below. For example: *All XXX files are available from the XXX database (accession number(s) XXX, XXX.*).<br>• If the data are all contained **within the manuscript and/or Supporting Information files**, enter the following: *All relevant data are within the manuscript and its Supporting Information files.*<br>• If neither of these applies but you are able to provide **details of access elsewhere**, with or without limitations, please do so. For example:<br><br>*Data cannot be shared publicly because of [XXX]. Data are available from the XXX Institutional Data Access / Ethics Committee (contact via XXX) for*	All relevant data are within the manuscript and its Supporting Information files.

researchers who meet the criteria for access to confidential data.<br><br>*The data underlying the results presented in the study are available from (include the name of the third party and contact information or URL).*<br>• This text is appropriate if the data are owned by a third party and authors do not have permission to share the data.<br><br>* typeset	
Additional data availability information:	Tick here if the URLs/accession numbers/DOIs will be available only after acceptance of the manuscript for publication so that we can ensure their inclusion before publication.

Powered by Editorial Manager® and ProduXion Manager® from Aries Systems Corporation

Click here to access/download;Manuscript;Mogaji et al.docx

Attendee Awareness and Knowledge about Preventive Chemotherapy Neglected Tropical Diseases (PC-NTD) control during the First World Neglected Tropical Diseases Day in Ekiti State, Nigeria.

Short title: Attendee awareness during the First World NTD Day in Ekiti

Hammed O. Mogaji[1*], Ikenna M. Odoh[2]., Concilia I. Iyeh[1]., Abdulhakeem A. Adeniran[3]., Segun I. Oyedeji[1]., Hilary I. Okoh[1]., Adedotun A. Bayegun[4]., Olaitan O. Omitola[4], Cynthia U. Umunnakwe[4]., Francisca O. Olamiju[5]., Olatunwa J. Olamiju[5] and Uwem F. Ekpo[4]

[1]Department of Animal and Environmental Biology, Federal University Oye-Ekiti, Ekiti State, Nigeria, [2]University Medical Centre, Federal University Oye-Ekiti, Ekiti State, Nigeria, [3]Laboratory of Molecular Biomedicine, Centre for Genomic, Biotechnology, Instituto Politecnico Nacional, Mexico, [4]Department of Pure and Applied Zoology, Federal University of Agriculture, Abeokuta, Ogun State, Nigeria [5]Mission To Save The Helpless (MITOSATH), Jos, Nigeria

* Corresponding author

Email: mogajihammed@gmail.com

Abstract

Background: The need to control Neglected Tropical Diseases (NTDs) and sustain progress towards elimination through mass administration of medicines requires substantial communal participation. This study, therefore, assessed the knowledge and perception of attendees' regarding NTDs and its control activities during the Inaugural World NTD day event in Ekiti State, Nigeria.

Methodology: A cross-sectional study involving the administration of pretested semi-structured questionnaires to consenting attendees at the Inaugural World NTD day event was conducted on the 30th January, 2020. The questionnaire collected data on attendee's demography, knowledge and awareness about NTDs in Nigeria. Quantitative data were analysed using descriptive statistics in SPSS. 20.0 Software and expressed as frequencies and percentages. However, qualitative data to support quantitative analysis were obtained using open-ended questionnaires and analysed thematically.

Principal findings/conclusion: A total of 309 attendees participated in this study comprising 167 (54.0%) females, and 142(46.0%) males. 167 (54.0%) of the attendees have not heard about NTDs before, whereas 77(24.92%) have heard about NTDs through the advertisement of the event. 105(33.98%) of the attendees were unaware of ongoing NTD control programs in schools and communities. Also, 246 79.61%) of them have not taken or do not know anyone that has taken drugs donated in schools or communities before. The number of attendees 41(13.3%) who incorrectly classified malaria as NTDs is higher than those who recognized onchocerciasis 36 (11.7%) and worm infections 34(11.0%) as NTDs ($p>0.05$). This study has shown that awareness and knowledge about NTDs control activities in Ekiti State is low, thus justifying the event as an awareness day

for addressing NTDs. Public enlightment and regular promotional activities such as media engagement will raise the public appreciation and participation in NTDs control activities.

Word count: 279

Authors summary

Neglected Tropical Diseases has remained a public health menace in most developing countries including Nigeria. Efforts targeted at controlling the diseases requires substantial community acceptance and participation in drug donation campaigns. This study, therefore, provides information on public knowledge and awareness about NTDs and its control activities. We surveyed 309 attendees during the inaugural 2020 World NTD Day event in Ekiti, Nigeria. Our results show that attendee knowledge and awareness about NTDs control programme and promotional activities is low. There is thus a need for NTD control programmers to engage in regular promotional activities such as media engagements to ensure improved awareness and community involvement.

Introduction

Neglected Tropical Diseases (NTDs) represents a diverse group of communicable, disabling, chronic and disfiguring diseases that prevail in tropical and subtropical conditions [1]. NTDs affect more than one billion people in 149 countries, most especially the rural and disadvantaged urban settings challenged by poverty. These areas are usually characterized by inadequate access to water and sanitation facilities, close proximity to infectious vectors and domestic animals or livestock [2-4]. There are propositions that NTDs could comprise as much as 40 different types of diseases [5], with only thirteen of them recognized initially [6]. These were later revised to 17 [7], and more recently to 20 with the addition of snakebite, scabies and other ectoparasites, mycetoma and chromoblastomycosis and other deep mycoses [1].

NTDs are more common in sub-Saharan Africa (SSA) with about 500 million people affected [3,4,6,8]. About 25% of Africa's NTD burden is in Nigeria [6,8]. Although the epidemiological profiles of snakebite, scabies and mycetoma remain largely unknown in Nigeria, over half of the WHO recognized NTDs are present in Nigeria [9-13]. The country also has the highest number of people infected or at risk in SSA with preventive chemotherapy NTDs (PC-NTDs) such as onchocerciasis, schistosomiasis, lymphatic filariasis and soil-transmitted helminths (STH) [6,8,14].

The 2012 London Declaration achieved a long-term commitment from pharmaceutical companies to donate essential medicines for the control of PC-NTDs in endemic countries, including Nigeria [15]. Donated medicines are delivered *en masse* to risk groups in schools

and households/communities via already established NTDs control programme architecture at sub-national/district levels [14]. This strategy known as mass administration of medicines (MAM) requires substantial commitment of both financial and human resources [16], and more importantly, substantial communal participation in the bid to sustain progress towards elimination targets [17-18]. However, there are pockets of evidence on the low public awareness about NTDs and efforts targeted at controlling it in Nigeria [13,19-22].

NTDs were one of the few health and developmental issues that didn't have a dedicated advocacy milestone. The World Health Organization has therefore set aside 30th January of each year as the annual World NTD Day to mobilize greater attention, action and investment on priority issues [23]. The annual World NTD day event therefore serves as an exciting opportunity to investigate the knowledge and perception of the public regarding NTDs and its control activities as it brought together stakeholders living or working in endemic countries and across the diverse NTD landscape. This study was therefore cconducted on 30th of January, 2020 during the Inaugural World NTDs day event held in Ekiti State, Nigeria

Methodology

Study area

This study was carried out in Ekiti State, Nigeria. Ekiti State is one of the six southwestern states in Nigeria. It has 16 Local Government Areas (LGAs) with Ado-Ekiti as the capital. The state is endemic for four major PC-NTD which includes Onchocerciasis, Lymphatic Filariasis, Schistosomiasis and Soil Transmitted Helminthiasis. MAM targeted at controling and possibly eliminating these diseases commenced in Ekiti State in 2015 with support from the non-governmental organizations, MITOSATH.

Planning and Preparatory Activities

As part of the Inaugural commemoration event for the World Neglected Tropical Diseases Day, a round-table stakeholders meeting was held on Thursday, 30th January 2020 at the Faculty of Science Auditorium, Federal University Oye-Ekiti. The event was well-advertised via a rally, live radio programme, and the web (email, Twitter, WhatsApp and Facebook). Invitation to attend was publicly announced to all interested members of the public.

Questionnaire Administration

The study was cross-sectional in design and involved the administration of already pretested semi-structured questionnaires to collect both quantitative and qualitative data from consenting participants. Participants were approached at the registration desk of the event and invited to participate in the study. Those who agreed to participate were given informed consent forms to complete after the purpose of the study has been explained to them. Information such as participants' demographic data, and their knowledge and

awareness about NTDs were assessed using the pretested questionnaires. To prevent event-induced responses and biases, attendees completed the questionnaires before proceeding with registration or gaining access to the event hall.

Data collection, processing, and analysis

Data obtained were entered into Microsoft Excel 2019 software. Quantitative data were analysed using descriptive statistics in SPSS. 20.0 Software and expressed as frequencies and percentages. However, qualitative data to support quantitative analysis were obtained using open-ended questionnaires and analysed thematically following standard data analysis steps.

Ethics Statement

This study received ethical approval from the Ethics Review Committees of State Primary Health Care Development Agency, Ekiti State, and Federal University Oye-Ekiti, Ekiti State, Nigeria (HRS/WNTD/001).

Results

Demographic information of study participants

A total of 309 attendees participated in the study, including 167 (54.0%) were females, and 142 (46.0%) were males. Also, the majority of the study participants 206 (66.7%) were within 15-25 years age category, and 4 (1.3%) participants were below 15 years of age. A total of 221 (71.5%) attendees were students or job-seekers, 48 (15.5%) work in tertiary educational institutions, 38 (12.3%) were health workers policymakers, 1 (0.3%) was a policy maker and 1 (0.3%) media personnel. (Table 1)

Table 1: Demographic characteristics of study participants

	**Number of respondents**	**Percentage**
**Sex**		
Male	142	46.0
Female	167	54.0
Total	309	100.0
**Age (in years)**		
<15	4	1.3
15-25	206	66.7
26-35	39	12.6
>35	60	19.4
Total	309	100.0
**Occupation**		
Students/unemployed	221	71.5
Academia*	48	15.5
Health professionals**	38	12.3
Policymakers	1	0.3
Media personnel	1	0.3
Total	309	100.0

**Academia was defined as anyone working in a tertiary educational institution including lecturers, researchers and administrative staff; **Health professionals was defined as anyone working in health institutions including hospitals, ministries of health, primary health care centers and is involved in the disease control programme and practice.*

Perception of attendees about NTDs as a disease and a public health problem

Among the attendees, 167 (54.0%) have not heard about NTDs before, while 110 (35.6%) have learnt about NTDs in the hospital and 77 (25%) have heard about NTDs through the advert of this event. Other sources of information listed were schools 18 (5.83%) and internet 15 (4.85%) (Table 1). Also, a total of 87 (28.2%) and 61 (19.7%) attendees were able to correctly identify schistosomiasis and lymphatic filariasis respectively as NTDs. Onchocerciasis and Soil-Transmitted Helminthiasis were also identified as NTDs by 36 (11.7%) and 34(11.0%) additional attendees respectively (Figure 1). Other significant diseases such as Malaria, Tuberculosis, HIV, and Diabetes were incorrectly listed as NTDs by 41 (13.3%), 27 (8.7%), 17 (5.5%) and 14 (4.5%) attendees respectively (Figure 1). 232 (75.0%) of the attendees think that NTD is a public health problem, with a total of 82 (26.5%) of the attendees ascribing several reasons for it (Table 1). 23 (28%) of the attendees believed that NTDs is a public health problem because it affects people in overcrowded communities and 21 (26%) ascribed it to the fact that poor awareness and low attention has been given to NTDs (Figure 2)

Table 2: Knowledge of study participants about NTDs

	Number of respondents	Percentage
**Have you heard about NTDs before ?**		
Yes	138	44.7
No	167	54.0
No response	4	1.3
Total	309	100.0
**Where did you learn about it?**		
Hospital	110	35.6
The advert of this event	77	24.92
School	18	5.83
Internet	15	4.85
No response	89	28.8
Total	309	100.0
**Do you think NTD is a public health problem?**		
Yes	232	75.08
No	30	9.71
No response	47	15.21
Total	309	100.0

Figure 1: Perceptions of attendees on what NTDs are

Figure 2: Perceptions of attendees on why NTDs is a public health problem

Perception of attendees about signs and symptoms of common NTDs

Of the 309 attendees, only 133 (43.04%) have seen a probable NTD case before. Among the 4 major symptoms of PC-NTDs selected for this study (bloody urine, swollen limbs, worm(s) in the stool and depigmented skin), a high proportion of the attendees 217 (70.23%), 166 (53.72%), 179 (57.93%) and 207 (66.99%) indicated that they have not seen anyone with bloody urine, swollen limbs, a worm in the stool and depigmented skin before the event respectively (Table 3)

Awareness about NTD control programming and promotional activities

A total of 181 (59%) of the attendees were aware of ongoing NTD control programmes in schools and communities. However, a high proportion 246 (79.61%) of them have not taken or do not know anyone that has taken drugs donated in schools or communities before (Table 4). Also, majority of the attendees 218 (70.6%) have not received health educational messages on NTDs before. Among the few that had received, the listed sources of information were in schools 26 (8.4%), hospitals 8 (2.6%), on radio programmes 4 (1.3%) and through the internet 2 (0.6%). Only 5 (1.6%) of the attendees think there is enough awareness about NTDs and majority of the participants 249 (80.6%) are willing to participate in NTD promotional activities including advocacy and health promotion 118 (38.2%), research 63 (20.4%) and networking 54 (17.5%). (Table 5).

Table 3: Awareness about signs and symptoms of NTDs

	Number of respondents	Percentage
**Have you seen anyone affected by NTDs before ?**		
Yes	133	43.04
No	166	53.72
No response	10	3.24
Total	309	100.0
**Have you seen anyone with swollen limbs or thigh before ?**		
Yes	137	44.34
No	166	53.72
No response	6	1.94
Total	309	100.0
**Have you seen anyone with bloody before ?**		
Yes	85	27.51
No	217	70.23
No response	7	2.26
Total	309	100.0
**Have you seen anyone with a worm in the stool before ?**		
Yes	120	38.83
No	179	57.93
No response	10	3.24
Total	309	100.0
**Have you seen anyone with depigmented skin before ?**		
Yes	88	28.48
No	207	66.99
No response	14	4.53
Total	309	100.0

Table 4: Awareness about NTDs control programme in schools and communities

	Number of respondents	Percentage
**Are you aware of any ongoing treatment programme for NTDs in schools or communities ?**		
Yes	181	58.58
No	105	33.98
No response	23	7.44
Total	309	100.0
**Do you know anyone that has taken the drug/medicine before?**		
Yes	49	15.86
No	246	79.61
No response	14	4.53
Total	309	100.0
**Do you know the name of the drug/medicine?**		
Albendazole	1	0.3
Coartem	1	0.3
Combantrin	1	0.3
Mectizan	8	2.6
Mectizan and Albendazole	1	0.3
Mectizan and Praziquantel	1	0.3
Praziquantel	1	0.3
Wormin	1	0.3
No response	294	95.1
Total	309	100.0
No response	14	4.53
Total	309	100.0

Table 5: Perception of attendees about public awareness on NTDs as a disease and other NTDs related activities

	Number of respondents	Percentage
**Have you received health educational messages on NTDs before?**		
Yes	67	21.68
No	218	70.55
No response	24	7.77
Total	309	100.0
**If yes, where did you receive such message(s)?**		
Community	5	1.6
Hospital	8	2.6
Internet	2	0.6
Ministry of Health	1	0.3
Radio	4	1.3
School	26	8.4
No response	241	78.0
Cannot remember	22	7.1
Total	309	100.0
**Generally, do you think there is enough awareness about NTDs and NTDs related activities?**		
Yes	5	1.62
No	55	17.80
No response	249	80.58
Total	309	100.0
**Are you willing to participate in any NTD promotional related activities?**		
Yes	249	80.58
No	37	11.97
No response	23	7.44
Total	309	100.0
**Which of the following are you willing to participate in?**		
Advocacy and health promotion (AHT)	118	38.19
Networking	54	17.48
Research	63	20.39
All of the listed	10	3.24
AHT and Networking	1	0.32
AHT and Research	1	0.32
No response	62	20.06

Discussion

Public awareness and perception about neglected tropical diseases are important factors in policy formulations, programme planning and modification of field practices focused at controlling NTDs [13,24]. The World NTD Day was recognized to call for action towards ending the burden of the NTDs via increasing advocacy for support, acknowledging strides, and increasing overall awareness about these group of debilitating diseases [5]. In this study, we assessed the awareness and perception of attendees at the inaugural World NTD Day event in Ekiti State, Nigeria. Findings showed that almost half of the attendees are unaware of NTDs before the event. This supports the existing notions that public awareness about NTDs is still low in Nigeria [19-22]. Furthermore, knowledge about NTD control programming and promotional activities is very poor, and this finding corroborates with that of [13].

More than 80% of the attendees do not know anyone who has benefitted from preventive chemotherapy mass drug administration and about 70% have not received any NTD health educational benefits prior this event. Ekiti is one of the rural State in Nigeria that has benefitted from the community-directed treatment with Ivermectin (CDTI) programme aimed at eliminating onchocerciasis since 2002 [25], and more recently from MAM since 2015 [14]. It is thus surprising that such a huge proportion of the attendees have not benefitted or heard about PC-NTD control programs. The role of residential proximities to health facilities had been sufficiently discussed by [26], as such our findings might have been influenced by the residential proximities of attendee' to areas where MAM were taking place. MAM are usually implemented in rural communities and quite impactful in endemic areas with less dynamic and diverse populations such as those found in rural areas compared to urban areas that are geographically larger in size and complex in human

population structure, making MAM more difficult to implement [27]. However, this study did not capture more specific information about attendee mobility and residential status to provide more explanations to the aforementioned low awareness about PC-NTD control programme.

On the other hand, sensitization and advocacy activities for NTDs are conducted once in a year, and usually very close to the set implementation dates for the annual MAM, with the focus of promoting compliance to medicine administration. This strategy offers a very limited time for intensive sensitization about NTDs [16], as compared to all year-round sensitization activities common to vaccination and bed-nets campaigns [28]. Communal support and awareness are heightened when repeated sensitization with community members, leaders and influential community structures, such as religious institutions, takes place [29]. Nevertheless, data from this study emphasizes the need for increased awareness on NTDs control activities and reiterates the importance of annual World NTD day as a platform for promoting NTD awareness and control activities. Interestingly, about 25% of the attendees indicated that they have heard about NTDs from the advert of the inaugural event. This, therefore, affirms the importance of open symposiums, online radio programme and rallies as important advocacy strategies in promoting health educational messages and intervention activities to end-users in common and public places such as schools, markets and hospitals [30].

It is not uncommon that an appreciable number of the attendees wrongly identified malaria as a neglected tropical disease compared to onchocerciasis and soil-transmitted helminthiasis. This can be attributed to the fact that there is a common ground of acceptance

for the morbidities and burden associated with malaria disease. The incubation period for *Plasmodium spp.*, the causative agent of malaria is between 7 and 30 days which is far shorter than the latency period for other NTDs such as onchocerciasis and soil-transmitted helminthiasis which may take several months or years [31]. This is reinforced by our findings where the majority of the attendees indicated that they have not seen anyone with bloody urine, swollen limbs, a worm in stool and depigmented skin before. This observation is in line with the findings of [18] and may contribute to the reasons why community members perceive less need for MAM over time. Also, unlike NTD control programmes, sustained malaria control programme have been in existence for quite a long period, and there are more resources commitments for interventions delivery such as distribution of insecticide treated bednets and intermittent preventive treatment to endemic regions than there are for NTDs control [32]. These might have contributed to more increased awareness for malaria control than most NTDs [13,33].

The WHO 2021-2030 NTDs roadmap clearly identified with awareness-generation activities aimed at educating and informing endemic communities on behavior changes, MDA scheduling, treatment and care options [34]. Without the public being mobilized to participate in NTD control and promotional activities in Nigeria, it may be increasingly difficult to meet set therapeutic coverage and achieve the desired objective of the WHO 2021-2030 NTDs roadmap of accelerating control and eventual elimination. This study has thus revealed a continuous low level of public perception of NTDs and their control activities as an earlier study in Nigeria also reported [13]. Efforts aimed at improving awareness on NTDs, disease control programs and health promotional activities should therefore be modified on this context to ensure optimal communal participation as these

diseases are as well endemic in urban settings [35]. Since sustainable promotional and awareness activities require substantial resources commitment and coordination [13,16,18]. Mass public health media campaigns about NTDs in urban areas will potentially improve public perception and contribute towards participation and uptake of NTDs intervention tools. However, in the absence of such, promotional activities that requires lesser financial commitments such as engagement with the media via online/TV interviews and podcasts should be done regularly to intimate the public about NTDs.

Conclusion

Public knowledge and awareness about NTDs control programme and promotional activities in Ekiti State, Nigeria is low. NTD control unit/department should be provided with adequate resources to implement regular promotional activities such as media engagement to intimate the public about NTDs.

Declarations

Consent for publication

Not applicable

Availability of data and materials

The datasets from the study are available and have been provided as supplementary file 1.

Competing interests

The authors declare that they have no competing interest

Funding

We express our deep appreciation to Crown Prince Court of Abu Dhabi for providing the micro-grant for the Inaugural World NTD day event in Ekiti State, Nigeria. However, funds for the research were provided by the first author, hence Crown Prince Court of Abu Dhabi had no role in funding this research, the study design, data collection and analysis, decision to publish, or preparation of the manuscript.

Authors' contribution

HOM and UFE conceptualized the study. HOM, IMO, AAA and OOO designed the protocol and research instrument. IMO, SIO, HIO, CII improved the protocol and pretested the instrument. CII, OOO, CUU, AAB participated in data collection. HOM performed all statistical analysis. HOM prepared the first draft of the manuscript, which was reviewed by UFE, SIO, OOO, AAA, FOO and OJO. All authors contributed to the development of the final manuscript and approved its submission.

Acknowledgement

We appreciate MITOSATH for donation of additional NTD posters and notebooks for attendees. We also appreciate the members of staff from the Neglected Tropical Diseases control unit, Ekiti State Primary Health Care Development Agency (SPHDA) for their support during the rally and live radio programme that precede this investigation. Finally, our heartfelt gratitude goes to Mrs Funmilayo S. Oluwafemi, Mrs Tunrayo Oluwadare, and all members of the local organizing committee from Federal University Oye-Ekiti and Afe Babalola University, Ado-Ekiti for their numerous support.

References

1. World Health Organization (WHO). Neglected Tropical Diseases. [Online]. 2020. Available at https://www.who.int/neglected_diseases/diseases/en/.
2. Hotez PJ, Molyneux DH, Fenwick A, Kumaresen J, Sachs SE, et al. Control of Neglected Tropical Diseases. *N Engl J Med.2007;*357(10):1018–27. https://doi.org/10.1056/NEJMra064142.
3. Qian MB, Zhou. XN. Global burden on neglected tropical diseases. *The Lancet Infect Dis. 2016;16*(10), 1113-1114.
4. Mitra AK, Mawson AR. Neglected Tropical Diseases: Epidemiology and Global Burden. *Trop Med Infect Dis*. 2017;2(3):36. Doi:10.3390/tropicalmed2030036
5. Hotez PJ, Aksoy S, Brindley PJ, Kamhawi S. What Constitutes a Neglected Tropical Disease?. *PLoS Negl Trop Dis*. 2020;14(1): e0008001. https://doi.org/10.1371/journal.pntd.0008001.
6. Hotez PJ, Kammath A. Neglected Tropical Diseases in Sub-Saharan Africa: Review of Their Prevalence, Distribution, and Disease Burden. *PLoS Negl Trop Dis*. 2009. https://doi.org/10.1371/journal.pntd.0000412.
7. World Health Organization (WHO). Neglected Tropical Diseases. [Online]. 2009. Available at https://apps.who.int/iris/bitstream/handle/10665/44214/9789241598705_eng.pdf?sequence=1.
8. Hotez PJ., Asojo AO, Adesina AM. Nigeria: 'Ground Zero' for the High Prevalence Neglected Tropical Diseases. PLoS Negl Trop Dis. 2012. https://doi.org/10.1371/journal.pntd.0001600.

9. Smith EC. Mycetoma in Nigeria. *Trans. R. Soc. Trop. Med. Hyg. 1928;*22(2),157–60. https://doi.org/10.1016/S0035-9203(28)90007-3.
10. Zarami BA, Nggada HA, Denue BA, Tahir MB. Clinical and Histological Characteristics of Mycetoma in a Tertiary Health Institution in Northeastern Nigeria. *Nig Hosp Prac*. 2015;16(1–3). https://doi.org/10.4314/NHP.V16I1-3.
11. Michael GC, Grema BA, Aliyu I, Alhaji MA, Lawal TO, et al. Knowledge of Venomous Snakes, Snakebite First Aid, Treatment, and Prevention Among Clinicians in Northern Nigeria: A Cross-Sectional Multicentre Study. *Trans R Soc Trop Med Hyg*. 2018;112(2). https://doi.org/10.1093/TRSTMH/TRY028.
12. Ugbomoiko US, Oyedeji SA, Babamale OA, Heukelbach J. 2018. Scabies in Resource-Poor Communities in Nasarawa State, Nigeria: Epidemiology, Clinical Features and Factors Associated with Infestation. *Trop. Med. Infect. Dis.* 3 (2). https://doi.org/10.3390/tropicalmed3020059.
13. Olamiju OJ, Olamiju FO, Adeniran AA, Mba IC, Ukwunna CC, et al. Public Awareness and Knowledge of Neglected Tropical Diseases (NTDs) Control Activities in Abuja, Nigeria. *PLoS Negl Trop Dis. 2014;*8(9). https://doi.org/10.1371/journal.pntd.0003209.
14. Federal Ministry of Health (FMOH). Nigeria NTD Master Plan (2015-2020). [Online]. 2015. Available from https://espen.afro.who.int/system/files/content/resources/NIGERIA_NTD_Master_Plan_2015_2020.pdf
15. Uniting to Combat NTDs. London Declaration on Neglected Tropical Diseases. 2012. Avaialble at https://unitingtocombatntds.org/london-declaration-neglected-tropical-diseases/.

16. Oluwole AS, Dean L, Lar L, Salami K, Okoko O, et al. Optimising the Performance of Frontline Implementers Engaged in the NTD Programme in Nigeria: Lessons for Strengthening Community Health Systems for Universal Health Coverage. *Hum Resour Health*. 17(1):79. https://doi.org/10.1186/s12960-019-0419-8.
17. Madon S, Malecela MN, Mashoto K, Donohue R, Mubyazi G, et al. The role of community participation for sustainable integrated neglected tropical diseases and water, sanitation and hygiene intervention programs: A pilot project in Tanzania. Soc Sci Med. 2018; 202:28-37. doi: 10.1016/j.socscimed.2018.02.016. PMID: 29501716; PMCID: PMC5906643.
18. Adekeye OB, Dean L, Dixon R Community Engagement in Neglected Topical Disease Treatment in Nigeria: Rethinking the needs of varying contexts. 2017. Available at https://countdown.lstmed.ac.uk/sites/default/files/content/centre_page/attachments/Community%20Engagement_final.pdf
19. Relief Web. Neglected Tropical Diseases not deserted, Governemnt collaborates with WHO to create awareness. [Online] 2020. Available at https://reliefweb.int/report/nigeria/neglected-tropical-diseases-not-deserted-government-collaborates-who-create-awareness
20. Gboeloh LB and Kingsley E. A Survey on Knowledge of Neglected Tropical Diseases (Ntds) Among Residence of Port Harcourt City, Rivers State, Nigeria. *Applied Science Reports.* 2018;19(3) Available at SSRN: https://ssrn.com/abstract=3201446
21. Wogu MD, Okaka CE (2008) The knowledge, attitude and perception of onchocerciasis and ivermectin treatment by the people in Okpuje, Edo State,

Nigeria. *Intern J Biomed Health Sci*. 4: 122–123.

22. Ugbomoiko US, Ofoezie IE, Okoye IC, Heukelbach J (2010) Factors associated with urinary schistosomiasis in two peri-urban communities in south–western Nigeria. *Ann Trop Med Parasitol*. 104: 409–419.
23. World NTD Day. [Online]. 2020. Available at https://worldntdday.org/results/
24. Mackey TK, Liang BA, Cuomo R, Hafen R, Brouwer KC, et al. Emerging and reemerging neglected tropical diseases: a review of key characteristics, risk factors, and the policy and innovation environment. *Clin Microbiol Rev*. 2014;27(4):949-979. doi:10.1128/CMR.00045-14
25. African Programme for Onchocerciasis Control (APOC). Report of the Twenty-fourth session of the Technical Consultative Committee (TCC) Ougadougou, 12-16 March 2007. DIR/COORD/APOC/REP/TCC24 09/05/2007. Available at https://www.who.int/apoc/about/structure/tcc/tcc24_final_report.pdf?ua=1
26. Kikuti M, Cunha GM, Paploski IAD, Kasper AM, Silva MMO, et al. Spatial distribution of dengue in a Brazilian urban slum setting: role of socioeconomic gradient in disease risk. *PLoS Negl Trop Dis.* 2015;9:e0003937.
27. Adams AM, Vuckovic M, Birch E, Brant TA, Bialek S, Yoon D, Koroma J, Direny A, Shott J, Lemoine JF, Dembele M, Baker MC. Eliminating Neglected Tropical Diseases in Urban Areas: A Review of Challenges, Strategies and Research Directions for Successful Mass Drug Administration. *Trop Med Infect Dis*. 2018 Nov 21;3(4):122. doi: 10.3390/tropicalmed3040122. PMID: 30469342; PMCID: PMC6306919.
28. World Health Organization (WHO). WHO Recommended Strategies for the Prevention and Control of Communicable Diseases. [Online]. 2001. Available at

https://apps.who.int/iris/bitstream/handle/10665/67088/WHO_CDS_CPE_SMT_2001.13.pdf

29. Omedo MO, Matey EJ, Awiti A, Ogutu M, Alaii J, Karanja DMS, Montgomery SP, Secor WE, Mwinzi PNM. Community health workers' experiences and perspectives on mass drug administration for schistosomiasis control in western Kenya: the SCORE Project. *Am J Trop Med Hyg*. 2012;87:1065–72.

30. Advocacy partnership. TB/MDR-TB Advocacy Tool Kit. [Online]. 2012. Available at http://www.stoptb.org/assets/documents/global/awards/cfcs/tb_mdr%20advocacy%20tool%20kit.pdf.

31. Center for Diseases Control and Prevention (CDC). Lifecycle [Online]. 2000 Available at https://www.cdc.gov/malaria/about/biology/index.html

32. Nabarro D. Roll Back Malaria. *Parassitologia.* 1999 Sep;41(1-3):501-4. PMID: 10697910.

33. Adeniran A, Mogaji O, Oluwole S, Abe M, Bankole O, et al. Commodity Utilization of Malaria Intervention and Control Tool: Access and Utilization of Long Lasting Insecticide Net in Rural Communities of Imeko-Afon and Odeda Local Government Area of Ogun State, Nigeria. *Ann Trop Med* **Public Health.** 2014;7(2). https://doi.org/10.4103/1755-6783.146382.

34. World Health Organization (WHO). Ending the Neglect to Attain Sustainable Development Goals: A roadmap for neglected tropical diseases 2021-2030. Available at https://www.who.int/neglected_diseases/Ending-the-neglect-to-attain-the-SDGs--NTD-Roadmap.pdf

35. Mogaji HO, Dedeke GA, Bada BS, Bankole S, Adeniji A, et al. Distribution of

ascariasis, trichuriasis and hookworm infections in Ogun State, Southwestern Nigeria. *PLoS ONE*. 2020;15(6). https://doi.org/10.1371/journal.pone.0233423

Click here to access/download;Figure;Fig 1.tiff

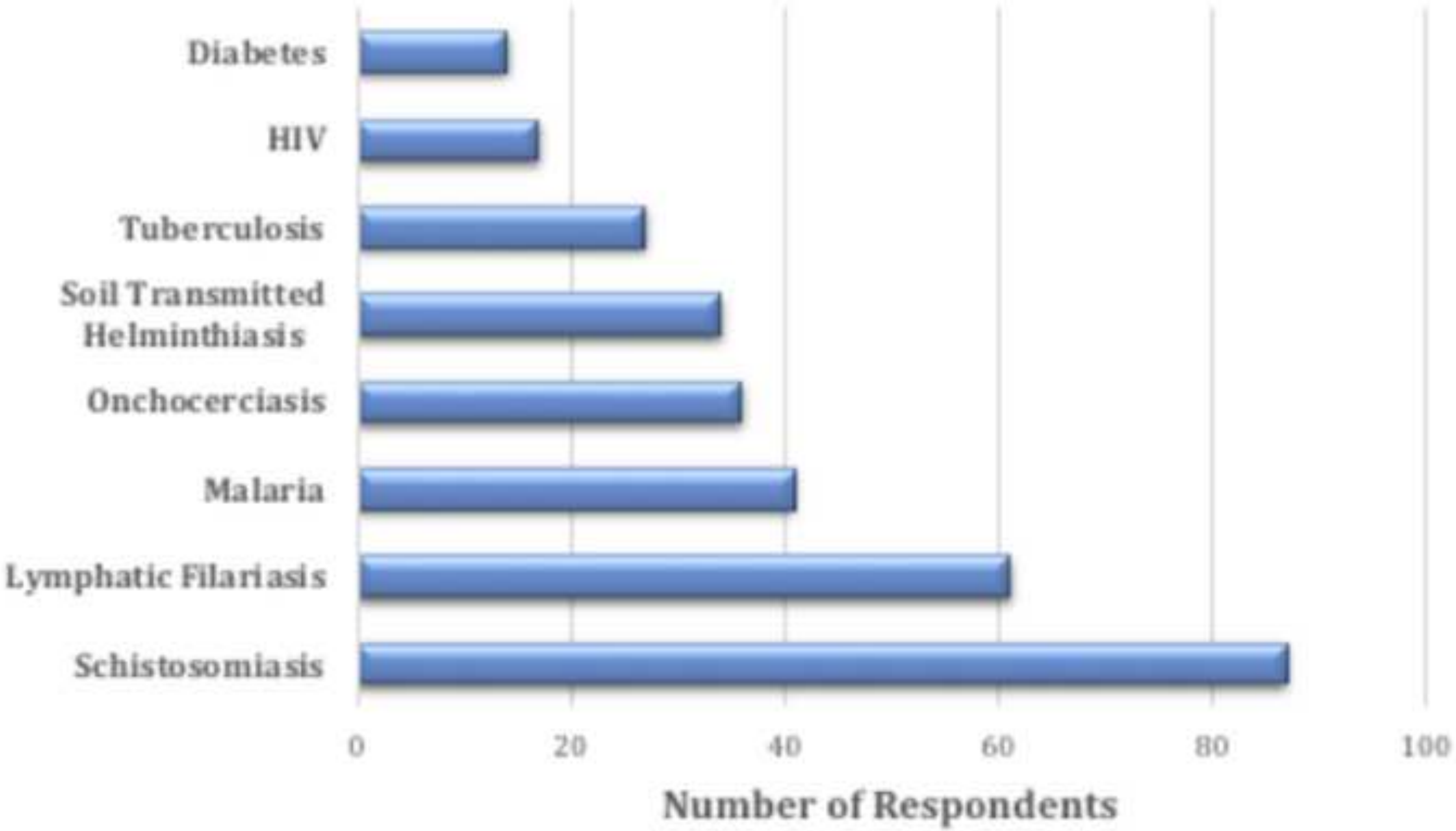

Click here to access/download;Figure;Fig 2.tiff

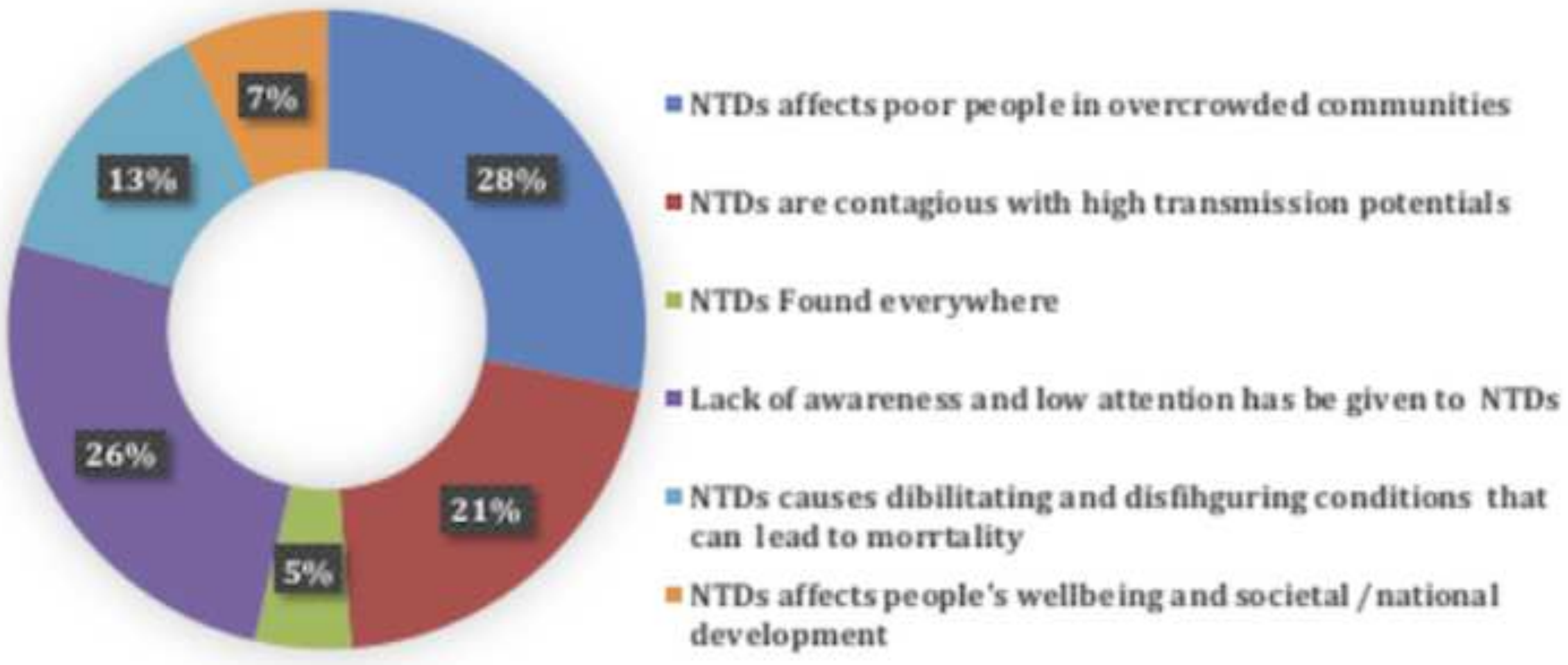

Click here to access/download
Supporting Information
Final Data (Autosaved).xlsx

Click here to access/download
Supporting Information
Mogaji_STROBE_checklist_cross-sectional.doc
