# Supplementary material for: Attendee’s awareness about preventive chemotherapy neglected tropical diseases (PC-NTD) control during the first world neglected tropical diseases day in Ekiti State, Nigeria
Source: PLoS Negl Trop Dis. 2021 Mar 29;15(3):e0009315. doi: 10.1371/journal.pntd.0009315 (PMC8032117; doi:10.1371/journal.pntd.0009315)
Supplement: Attachment — Submitted filename: RESPONSE TO REVIEWER 2.docx [file pntd.0009315.s005.docx]

**RESPONSE TO REVIEWER 2:**

Comment 1: The objectives of the study are not well stated. The study protocol is faulty
The population and sample size are not adequate for the study and the statistical analysis are too elementary.

R1: Thank you very much for your very wonderful comments and revision, which has improved our manuscript. We understand the analysis were too elementary, so we have performed additional statistical analysis to address the concerns raised here. We extracted the occupational status of the attendees and performed Chi—square tests to check for associations. Please see all modified tables 2-5 and accompany text within Lines 201-282

Also, thank you very much for raising the comments on the objectives of the research which was not well stated before now. We completely agree with you and we like to state this must have been an omission.

Please find the objective included in between lines 103-105.

Line 103-105: This study therefore aims to investigate public awareness about NTDs and efforts targeted at controlling it in Ekiti State, where such information is lacking.

In addition, we have included a new sub-section on limitations to discuss the gaps in our study protocol, and the usefulness of our data and of course the limits.

Line 372-390:

**Limitations**

This study is a rapid assessment of Attendee’s awareness during the maiden World NTD Day event in Ekiti State. The study has the following limitations; (1) the convenient sampling methodology employed in recruiting study participants contributed to the low sample size and restricted sampling frame, (2) to avoid interfering with attendee’s participation in the event, data collectors do not have enough time to engage and probe attendees adequately, which might have led to the non-response rate recorded for some variables in the questionnaire (3) this study did not capture more specific information on attendee mobility and residential status that could provide more explanations to the low awareness about PC-NTD control programme observed.

Based on these limitations, our results are descriptive cannot be used to make inference on the large populace in Ekiti State. However, this study has highlighted some important concepts on awareness and community engagement in NTD control programming. This study therefore offers useful information and research gaps that should be explored using a more robust sampling methodology. More importantly, there is need to repeat the same knowledge assessment in rural communities using a more systematic sampling approach, utilize focus group discussions and avail the questionnaires in a language that is accessible to rural communities to ensure they can express themselves fully.

Comment 2: The analysis conforms with the results presented

R2: Thank you very much for this comment. We really appreciate

Comment 3: The conclusion is vague due to inadequate and inappropriate sample size

R3: Thank you very much for this comment. We have reworked our manuscript completely. Many thanks to your comments and that of the first reviewer. We now have modified results, discussion and as well conclusion that speaks to the data at hand.

Please find the conclusion between lines 349-399

**Line 394-399:**

**Conclusion:**

Public knowledge and awareness about NTDs control programme and promotional activities among attendees of the Maiden World NTD Day event in Ekiti State, Nigeria is low. Investments in regular radio programmes, community dialogues, and massive public health media campaigns targeted at improving public perception on NTDs and contributing towards uptake of NTDs intervention and participation in NTDs promotional activities should be explored.

Comment 4: The manuscript though interesting has some major drawbacks.
-the objectives of the study are not well defined

R4: Also, thank you very much for raising the comments on the objectives of the research which was not well stated. We completely agree with you and we like to say this must have been an omission.

Please find the objective included in between lines 103-105.

Line 103-105: This study therefore aims to investigate public awareness about NTDs and efforts targeted at controlling it in Ekiti State, where such information is lacking.

Comment 5: -the map or coordinates of the site or state is not shown or given

R5: We have now produced a new figure showing the study location.

**Figure 1: Map showing the study area with Nigeria as inset**

Comment 6: -the authors did not state how the 16 LGAs of the state were mobilized,
-the poor mobilization accounted for the poor sampling frame as shown by the demographic status of the respondents, where most of them where students. PC-NTDs are mostly targeted at rural communities where the disease burden is mostly found and noticed. Urban setting as reflected in this study is a wrong and biased sampling approach

R6: Thank you very much for your valuable comments. We have reworked our manuscript to provide more clarity.

Firstly, Ekiti state, comprises of 16 LGAs, 13 of which are rural. And the study was carried out in Oye, one of the rural LGAs. Sensitization and invitation to the event where the study took place was done via a rally and on a live radio programme with coverage across the state.

Line 136-139: The event was well-advertised via a rally, live radio programme, and the web (email, Twitter, WhatsApp and Facebook). Invitation to attend was publicly announced to all interested members of the public.

Line 326-331: Interestingly, about 25% of the attendees indicated that they have heard about NTDs from the advert of the inaugural event which included a rally and live broadcast on a radio channel. This, therefore, reiterates the potential of open symposiums, online radio programme and rallies as important advocacy strategies in promoting health educational messages and intervention activities to end-users in common and public places such as schools, markets and hospitals [31].

However, we agree that the convienient sampling approach used in this study will only allow us include those who have the capacity (health, wealth, time etc) to attend the event. We consider this as a major limitation. And we have described extensively in our new subsection what our limitations are. However, we still consider that some of our findings are useful and can stimulate further investigations in the rural community using a systematic sampling approach.

We must thank you very raising this very critical comment. Which has improved the quality of our manuscript.

Comment 7: -the questionnaire is faulty and wrongly framed e.g question 2 in table 2 should be "if yes,where.....table3 (have you seen any one with worm in the school) how does the authors expect the respondents to know except they have been inspecting stools in their communities. most of the questions are too ambiguous

R7: Thank you very much for your valuable comments. We have included the questionnaire as a supplementary file. The data collectors never had any issues while collecting the data from the attendees. However, we have reworked our manuscript to provide more clarity. And included language barrier as part of our limitation. We hope to work on these limitations as we plan for a future study using a more robust sampling approach.

Comment 8: -data analysis is too elementary to warrant a meaningful discussion

R8: Thank you very much for your very wonderful comments and revision, which has improved our manuscript. We understand the analysis were too elementary, so we have performed additional statistical analysis to address the concerns raised here. We extracted the occupational status of the attendees and performed Chi—square tests to check for associations. Please see all modified tables 2-5 and accompany text within Lines 201-282
